# Supplementary material for: Detection of Antibody Responses Against SARS-CoV-2 in Plasma and Saliva From Vaccinated and Infected Individuals
Source: Front Immunol. 2021 Dec 20;12:759688. doi: 10.3389/fimmu.2021.759688 (PMC8721203; doi:10.3389/fimmu.2021.759688)
Supplement: Supplementary file 2 [file DataSheet_2.pdf]

Supplemental Table 1

(A)

Vaccinated subjects				
**ID**	**Age**	**Sex**	**Days post-vaccination**	**Vaccine**
**RN#1-6**	55-59	F	29	Pfizer
**RN#4-2**	60-64	F	23	Pfizer
**RV#1**	60-64	M	22	Moderna
**RV#2**	60-64	M	20	Moderna
**RV#3**	65-69	M	15	Moderna
**RV#4**	50-54	F	15	Moderna
**RV#5**	50-54	M	37	Moderna

(B)

Convalescent COVID-19 patients				
**ID**	**Age**	**Sex**	**Days post symptom onset**	**Disease severity**
**RP#2-2**	50-54	F	>189	Asymptomatic
**RP#3-2**	55-59	M	226	Ambulatory
**RP#4-3**	60-64	M	210	Hospitalized
**RP#5-3**	60-64	M	225	Hospitalized
**RP#7-2**	40-44	F	243	Ambulatory
**RP#12**	25-29	M	256	Ambulatory
**RP#13**	25-29	F	246	Ambulatory

Supplemental Table 2

(A)

Vaccinated subjects				
**ID**	**Age**	**Sex**	**Days post-vaccination**	**Vaccine**
**70771**	35-39	M	27	Pfizer
**39325**	30-34	F	25	Pfizer
**54133**	60-64	M	28	Pfizer
**60982**	35-39	M	25	Pfizer
**66685**	30-34	F	29	Pfizer
**83690**	30-34	F	26	Pfizer
**31471**	30-34	F	30	Pfizer
**85708**	30-34	M	24	Moderna
**85971**	60-64	F	30	Pfizer
**46611**	30-34	M	30	Pfizer
**82596**	35-39	M	31	Pfizer
**82469**	>=65	M	30	Pfizer
**97478**	35-39	F	28	Moderna
**61521**	45-49	M	27	Moderna
**19492**	30-34	F	27	Pfizer
**53518**	30-34	F	28	Moderna
**61701**	30-34	F	29	Pfizer
**11150**	60-64	F	28	Moderna
**53676**	25-29	F	30	Moderna
**65670**	40-44	F	29	Pfizer

(B)

Convalescent COVID-19 patients				
**ID**	**Age**	**Sex**	**Days post symptom onset**	**Disease severity**
**CVAP1**	40-44	M	177	moderate
**CVAP3**	70-74	M	189	severe
**CVAP4**	60-64	M	172	mild
**CVAP13**	50-54	M	206	mild
**CVAP14**	55-59	M	207	mild
**CVAP20**	65-69	M	218	mild
**CVAP23**	70-74	M	229	moderate
**CVAP24**	75-79	M	216	none
**CVAP31**	65-69	M	214	mild
**CVAP32**	55-59	M	184	mild
**CVAP38**	75-79	M	205	mild
**CVAP40**	40-44	M	199	severe
**CVAP41**	55-59	M	134	mild
